# Supplementary material for: Precise atom-to-atom mapping for organic reactions via human-in-the-loop machine learning
Source: Nat Commun. 2024 Mar 13;15:2250. doi: 10.1038/s41467-024-46364-y (PMC10937625; doi:10.1038/s41467-024-46364-y)
Supplement: Supplementary file 1 — Supplementary Information [file 41467_2024_46364_MOESM1_ESM.pdf]

Supplementary information

Precise Atom-to-Atom Mapping for Organic Reactions via human-in-the-loop machine learning

Shuan Chen[1,2], Sunggi An[1,2], Ramil Babazade[3], Yousung Jung[1,2*]

1. Department of Chemical and Biomolecular Engineering, KAIST, 291 Daehak-ro, Daejeon 34141, South Korea
2. Department of Chemical and Biological Engineering, Seoul National University, 1 Gwanak-ro, Gwanak-gu, Seoul, 08826, South Korea
3. Graduate School of AI, KAIST, 291 Daehak-ro, Daejeon 34141, South Korea

*Correspondence: yousung.jung@snu.ac.kr

S1. Extended local reaction template (ELRT)

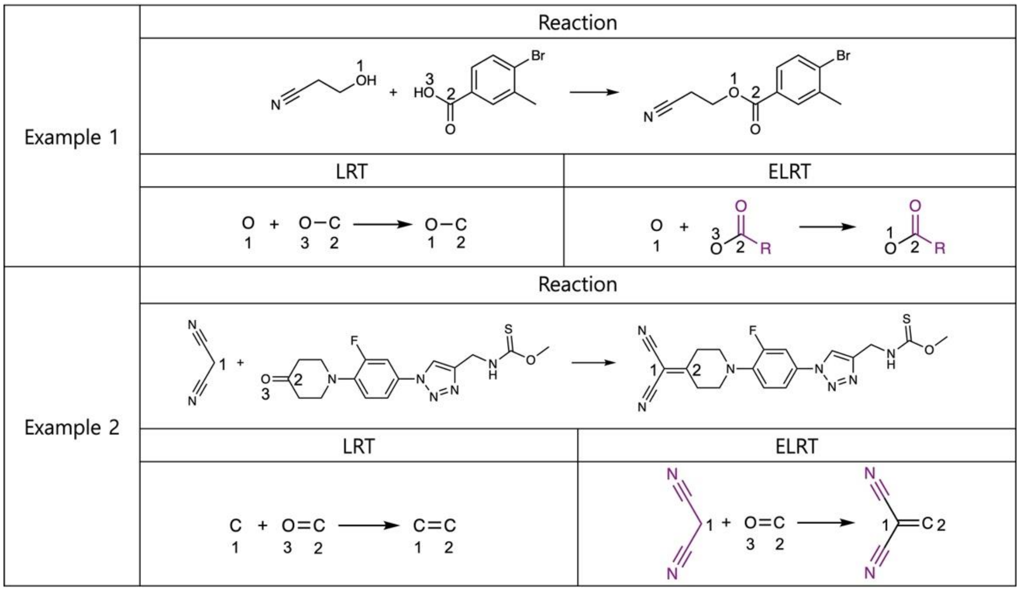

Supplementary Figure S1. Two examples showing the difference of expressive power between local reaction template (LRT)[1] and extended-local reaction template (ELRT). The original LRT cannot clearly express the chemical reactions, where the ELRT include the important part of the reactions (highlighted in purple), such as carbonyl groups and nitrile group.

Supplementary Table S1. The SMARTS of fragments used to extend the local reaction templates.

Fragment SMARTS	Description
**Functional groups**	
[OH0,SH0]=C-O	carbonyl acid
[C,O,N]=[C,O,N]	common double bond
[C,N]#[C,N]	alkyne/nitrile
O-C-O	acetal group
**Specific acid environment (colored in purple)**	
[*]=[*]-[C;X4;!$([CX4][F,Cl,Br,I,OH])]	acidic carbon
a-[C;X4;!$([CX4][F,Cl,Br,I,OH])]	acidic carbon
[*]#[*]-[C;X4;!$([CX4][F,Cl,Br,I,OH])]	acidic carbon
**Aryl oxygen (colored in purple)**	
a-O	adjacency to aromatic ring
a-C-O	one step from aromatic ring

S2. The problematic reactions in USPTO-50K dataset

To fairly evaluate the AAMs of the evaluated models on USPTO-50K dataset, we filter out reactions with by two criteria:

1. *Invalid product map*: products having duplicated or missing atom-map (347 reactions)
2. *Confusing reagent:* existence of product-like reagents (Tanimoto similarity >= 0.5, 819 reactions)

We show three examples for each type of the problematic reactions in Supplementary Figure S2. More examples can be found in https://github.com/snu-micc/LocalMapper/blob/main/data/USPTO_50K/view_problematics.ipynb.

Example reaction	Comment
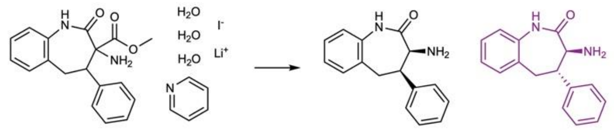	Invalid product map
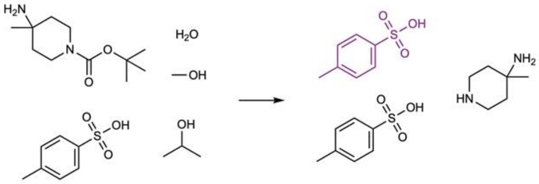	Invalid product map
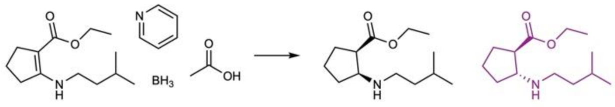	Invalid product map
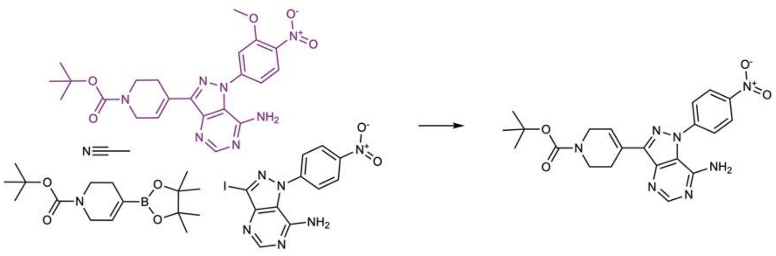	Confusing reagent
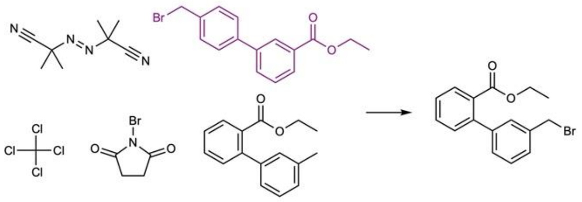	Confusing reagent
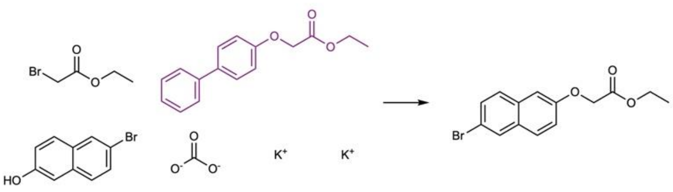	Confusing reagent

Supplementary Figure S2. Examples of reactions with invalid product and confusing reagent in the USPTO-50K dataset. The molecules causing the problems are highlighted in purple.

S3. The problematic reactions in the golden dataset

We show three examples of the unbalanced reactions recorded in the golden dataset[2] in Supplementary Figure S3. Full problematic reactions can be found in https://github.com/snu-micc/LocalMapper/blob/main/data/Golden/view_problematics.ipynb.

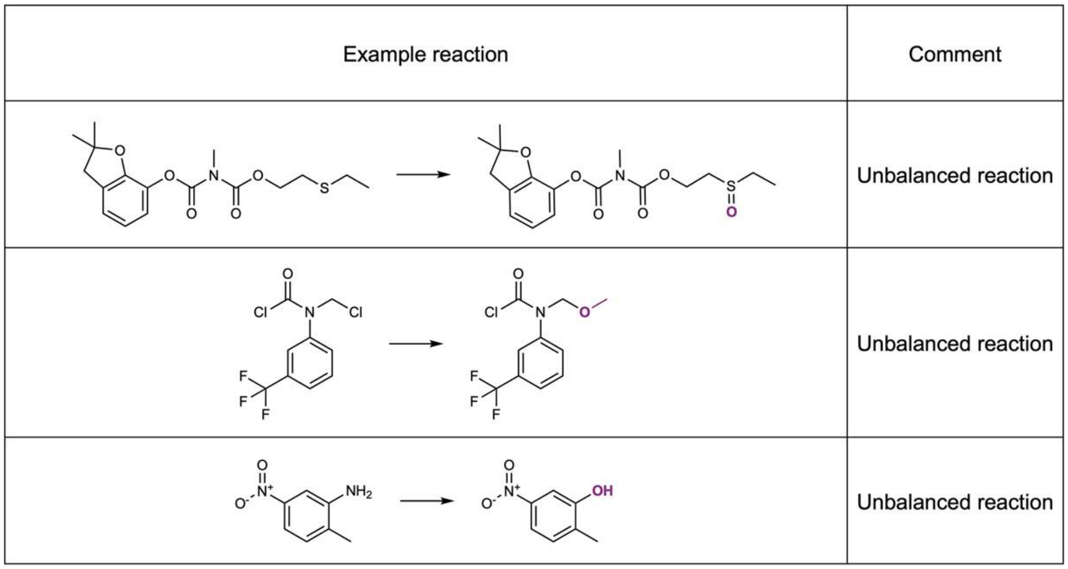

Supplementary Figure S3. Examples of unbalanced reactions in the golden dataset. The atoms causing the problems are highlighted in purple.

S4. The incorrectly mapped reactions in the golden dataset

Reaction index	Example reaction	Comment
481	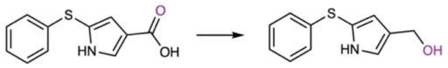	Wrong AAMs
698	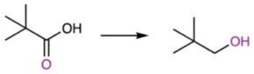	Wrong AAMs
1406	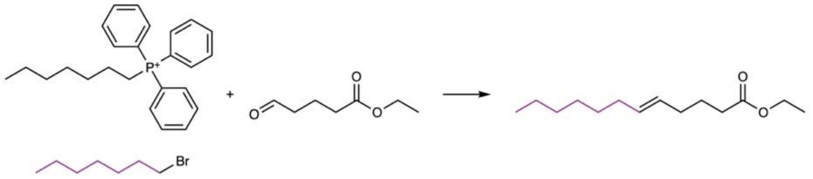	Wrong AAMs
1514	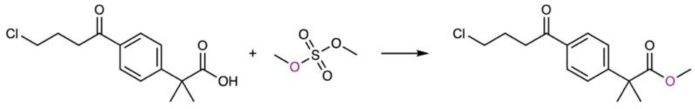	Wrong AAMs
1577	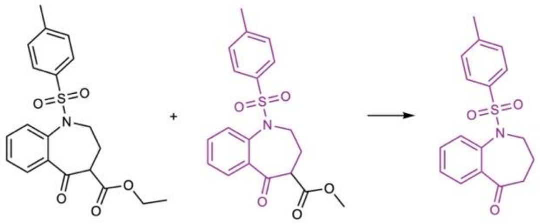	Selective reaction
1617	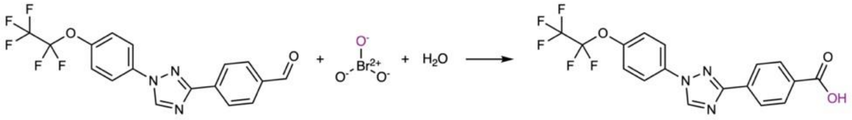	Wrong AAMs
1657	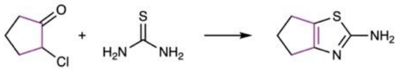	Wrong AAMs
1689	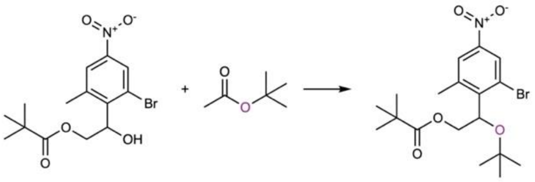	Wrong AAMs

Supplementary Figure S4. Examples of incorrectly mapped reactions in the golden dataset. The molecules without atom-mapping are removed for reading clarity, and the atoms causing the problems are highlighted in purple.

Supplementary Table S2. AMM results of RXNMapper, GraphormerMapper, and LocalMapper on manual-mapped reactions examined on four different sources following original AAMs. The highest accuracy and ratio are highlighted in bold font.

Model	Golden	USPTO	Typical	Complex
		Accuracy of all predictions		
RXNMapper[18]	86.1%	89.5%	91.9%	58.1%
GraphormerMapper[26]	82.8%	93.8%	88.4%	**66.1**%
LocalMapper (this work)	**89.1**%	**99.2**%	**93.1**%	**66.1**%
		Accuracy (ratio) of confident predictions		
RXNMapper[18]	95.1% (19.7%)	90.2% (23.8%)	95.7% (13.3%)	50.0% (**12.9**%)
LocalMapper (this work)	**99.1**% (**53.3**%)	**100**% (**79.7**%)	**100**% (**42.8%**)	**100**% (6.5%)

S5. Statistics of ELRTs extracted from USPTO-50K dataset

Supplementary Table S3. Top 10 popular ELRTs in the USPTO-50K dataset (mapped by Indigo[3])..

Rank	Extended-local reaction template	Description	Ratio
1	[N:2].O-[C:1]=O>>O=[C:1]-[N:2]	Nucleophilic acyl substitution	11.1%
3	[N:1].Cl-[c:2]>>[N:1]-[c:2]	SNAr	4.8%
4	C-C(-C)(-C)-O-C(=O)-[N:1]>>[N:1]	Nucleophilic acyl substitution	3.8%
5	[N:2].Cl-[C:1]=O>>O=[C:1]-[N:2]'	Nucleophilic acyl substitution	3.7%
6	[N:1].O=[C:2]>>[C:2]-[N:1]	Reductive amination	3.4%
7	O=[N:1]-[O-]>>[N:1]	Nitro group reduction	3.2%
8	[N:1].Br-[C:2]>>[C:2]-[N:1]	SN1/SN2	2.4%
9	Br-[c:1].O-B(-O)-[c:2]>>[c:1]-[c:2]	Suzuki coupling	2.2%
10	[N:1].Cl-[S:2]>>[N:1]-[S:2]	SN1/SN2	2.1%

Supplementary Table S4. Statistics of ELRT in the USPTO-50K dataset (mapped by Indigo[3])

Category	USPTO-50K dataset
# reactions	49,996
# ELRTs	1,102
Avg. # reaction/ELRT	45.37

S6 Atom and Bond Featurization

Supplementary Table S5. The chemical properties for initializing atom features and bond features.

Feature type	Chemical information
Atom features	atom types, chirality, formal charge, partial charge, aromaticity, hybridization, hydrogen bond donor/acceptor, the number of rings the atom belongs to
Bond features	Bond type, bond conjugation, bond in ring, stereo configuration

Supplementary references

1. Chen, S. & Jung, Y. Deep Retrosynthetic Reaction Prediction using Local Reactivity and Global Attention. *JACS Au* **1**, 1612–1620 (2021).
2. Lin, A. *et al.* Atom-to-atom Mapping: A Benchmarking Study of Popular Mapping Algorithms and Consensus Strategies. *Molecular Informatics* **41**, 2100138 (2022).
3. Indigo Toolkit (2024). https://lifescience.opensource.epam.com/indigo/ (accessed 23 Jan 2024).
